# Brief antibiotics exposure during adolescence produces persistent effects on the microbiome and fentanyl-seeking during adulthood in rats

**DOI:** 10.3389/fnbeh.2026.1916554

**Published:** 2026-10-12

**Authors:** Rebecca S. Hofford, Ava L. Shipman, Weiwei Wang, TuKiet T. Lam, Drew D. Kiraly

**Affiliations:** 1 Department of Psychiatry, Icahn School of Medicine at Mount Sinai, New York, NY, United States; 2 Friedman Brain Institute, Icahn School of Medicine at Mount Sinai, New York, NY, United States; 3 Yale/NIDA Neuroproteomics Center, Yale University, New Haven, CT, United States; 4 Keck MS and Proteomics Resource, Yale School of Medicine, New Haven, CT, United States; 5 Department of Molecular Biophysics and Biochemistry, Yale University, New Haven, CT, United States

**Keywords:** adolescence, drug seeking behavior, fentanyl, microbiome, self-administration (SA)

## Abstract

**Introduction:**

Adolescence is a developmentally sensitive period during which individuals begin experimenting with drugs of abuse. This window coincides with maturation of brain structures critical to reward and decision-making, including the prefrontal cortex, as well as peripheral physiological systems such as the gut microbiome - making it a period of heightened vulnerability to the long-lasting effects of drug exposure. Work from our lab has shown that manipulations to the gut microbiome influence drug reward, reinforcement, and drug seeking after abstinence. Critically, some of these effects are age-dependent with adolescents demonstrating greater sensitivity to the effects of microbiome disturbance on drug reward. Behavioral changes induced by microbiome disruption occur in parallel with molecular changes in reward-related brain regions like the medial prefrontal cortex. However, whether adolescent microbiome disruption produces alterations in opioid seeking and molecular changes in brain reward regions that persist into early adulthood has not been investigated.

**Methods:**

In the current study, we examined the effects of oral antibiotics during adolescent fentanyl self-administration on drug seeking, microbiome composition, and proteomic changes in the prefrontal cortex in adulthood.

**Results:**

Adolescent antibiotics reduced contextual but not cue-induced drug seeking and changes in the medial prefrontal cortex proteome suggest global alterations in pathways regulating cellular metabolism and neurotransmitter release.

**Discussion:**

Together this work indicates that brief oral antibiotics during adolescence produces long-lasting impacts on both brain and behavior.

## Introduction

Adolescence is often considered a sensitive period for the development of substance use disorders (SUD). Not only is this the time of life when most individuals initiate drug use (Alcover and Thompson, 2020), drug experimentation at young ages is associated with an increased lifetime risk of developing an SUD (King and Chassin, 2007; Chen et al., 2009). The reasons for adolescents’ vulnerability are multifaceted but include both environmental and physiological factors. Understanding how environmental perturbations occurring during adolescence influence drug taking and long-term consequences are necessary to help reduce the number of individuals developing an SUD.

One contributing factor to adolescents’ increased vulnerability is their ongoing development of the prefrontal cortex (Larsen and Luna, 2018). This region mediates higher cognitive functions such as attention, decision-making, and emotional regulation (Blakemore and Robbins, 2012; Etkin et al., 2015). At this time of life, this region undergoes drastic functional and structural changes such as synaptic pruning and refinement of inhibitory microcircuits (Mallya et al., 2019; Juraska and Drzewiecki, 2020; Caballero et al., 2021). This region is also considered an integral part of the mesocorticolimbic reward pathway (Cooper et al., 2017). Broadly, the role of the prefrontal cortex in drug taking and seeking is complex but dysfunction in this region is associated with greater risk of relapse (Goldstein and Volkow, 2011). In animal models, disruptions to medial prefrontal cortex (mPFC) alter rates of extinction and affect responding during reinstatement and drug-seeking after abstinence (Di Pietro et al., 2006; Moorman et al., 2015).

In addition to a developing mPFC, other peripheral body systems are undergoing marked changes during adolescence, including the gut microbiome. Adolescents have a microbiome that is distinct from both children and adults (Agans et al., 2011; Hollister et al., 2015) and this difference has been proposed to modulate neuropsychiatric outcomes (McVey Neufeld et al., 2016). In rodent models, disruption of the gut microbiome during adolescence produces behavioral effects that differ from adults (Hofford et al., 2025). In other studies, disruption to the microbiome during adolescence produces long-lasting impacts on intestinal health (Al Nabhani et al., 2019), hypothalamic-pituitary-adrenal axis function (Sudo et al., 2004), and anxiety-like behavior into adulthood (Desbonnet et al., 2015; Osman et al., 2023).

Previous work from our lab has demonstrated that knockdown of the microbiome with oral antibiotics (Abx) alters cocaine and opioid reward, reinforcement, and drug-seeking after abstinence in adult animals (Kiraly et al., 2016; Hofford et al., 2021, 2024; Meckel et al., 2024; Dave et al., 2025). Specifically, Abx reduces morphine conditioned place preference (CPP) (Hofford et al., 2021), increases fentanyl self-administration at higher fixed ratios, and fentanyl seeking after abstinence (Hofford et al., 2024). Interestingly, we observed that adolescents but not adults had reduced morphine CPP after only 5 days pretreatment with Abx (Hofford et al., 2025). The reduction in morphine CPP observed in adolescents on Abx was similar in magnitude to that seen in Abx adults after 14 days pretreatment, suggesting that Abx produces similar effects in adolescents and adults but that adolescents are more sensitive to these effects.

In conjunction with the behavioral results, Abx altered opioid-induced gene expression in the mPFC, and this differed between adolescents and adults. Additionally, the baseline age difference in gene expression within mPFC was quite robust. However, Abx and combination Abx and morphine drastically altered this gene expression profile, suggesting disruption of the microbiome at this age can alter developmental gene expression profiles in mPFC (Hofford et al., 2025). However, we do not know if the changes in gene expression observed in adolescents after combination Abx and opioid have any lasting consequences on neurobiology or behavior. In the current study, we restricted adolescent Abx exposure to coincide with fentanyl self-administration. We returned rats to normal H_2_O during abstinence before determination of changes in fentanyl seeking, microbiome composition, and proteomic changes in the mPFC during young adulthood.

## Materials and methods

### Animals

Male and female Sprague-Dawley rats (Envigo) were pair-housed with a sex-matched peer upon arrival to the colony on PND 22. The colony room was reverse light:dark cycle (lights on 1900) and was temperature and humidity controlled. All animal procedures were approved by the IACUC committee at Mount Sinai School of Medicine and all procedures adhered to ARRIVE guidelines.

### Drugs

Fentanyl hydrochloride was obtained through the NIDA drug supply program and was dissolved in saline at a stock concentration of 8 ug/ml. Individual self-administration syringes were further diluted to a final dose of 0.8 ug/kg/infusion.

### Drink solutions

Rats were placed on control H_2_O or a cocktail of Abx on PND 31. Abx consisted of 0.5 mg/mL vancomycin (Chem-Impex International), 2 mg/mL neomycin (Fisher Scientific), 0.5 mg/mL bacitracin (Research Products International), and 1.2 microg/mL pimaricin (Infodine Chemical) dissolved in water. Rats remained on drink solutions for five days before the start of self-administration and remained on their drink solutions throughout self-administration. On the day following their last self-administration session, all rats were returned to H_2_O for the remainder of the experiment.

### Fentanyl self-administration

Self-administration and the fentanyl-seeking test occurred in standard rat operant chambers with 2 levers, two jewel lights above each lever, a house light, and a syringe pump (Med Associates, St Albans VT). All rats were implanted with a jugular catheter under ketamine/xylazine anesthesia (100/10 mg/kg) between PND 29-31. Jugular catheters (VABR1B/22, Instech, Plymouth Meeting PA with silicone tubing, DuPont Liveo) were inserted into each rat’s jugular vein, secured to the vein with silk thread, and threaded under their skin to exit their body via a back mount. Rats were allowed to recover individually until they regained consciousness before returning to pair housing. After six-eight days recovery period, rats began self-administration.

Starting on PND 36, rats were trained to self-administer 0.8 ug/kg/infusion fentanyl on a fixed-ratio 1 schedule of reinforcement. Self-administration lasted only 5 days to ensure rats stayed within the adolescent period for the entirety of self-administration. Self-administration sessions occurred twice daily (Cho et al., 2020; Olekanma et al., 2023) and lasted 1.5 h during the rats’ dark phase. Sessions on the same day occurred three hours apart (experimental timeline in Figure 1A). During self-administration, completion of the ratio requirements resulted in a 5.9 s infusion and concurrent illumination of the light above the active lever that remained illuminated after infusion for a total of 20 s. During this time, no additional infusions could be earned. Active and inactive lever placement was counterbalanced across all rats. All rats’ active levers were baited with one operant food pellet (Bio-Serv Precision Reward Pellets for rodents) to encourage lever pressing on session 5 only; no lever baiting occurred after session 5. Food was taken away from all rats 24 h before the start of acquisition and before the fentanyl-seeking test; otherwise, rats were fed 14 g standard chow/rat during active self-administration and had *ad libitum* food access before self-administration started and during abstinence.

**Figure 1:**
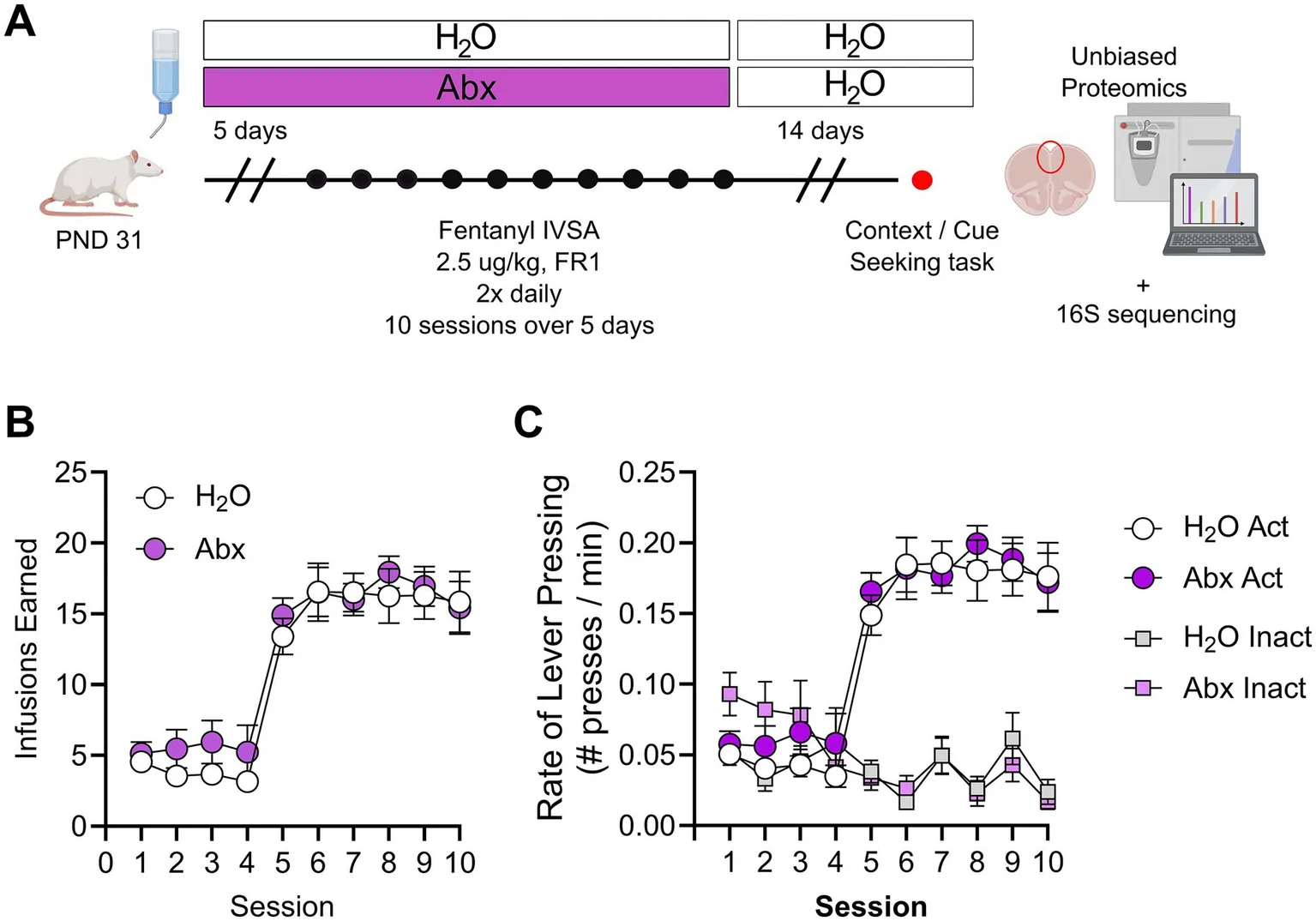
Brief Abx during adolescence does not affect fentanyl self-administration. **(A)** Experimental timeline. **(B)** Abx did not affect the number of infusions earned during self-administration. **(C)** Abx did not affect active lever press rate during fentanyl self-administration. Act, active lever; Inact, inactive lever.

All rats underwent 14 days of home cage abstinence followed by a combined context + cue fentanyl-seeking test. For this task, rats were placed in the operant boxes with both levers extended. Lever presses were recorded but had no programmed consequence for 30 min; this is referred to as the context portion of the seeking task. To signal the start of the cue portion of the seeking task, the light above the active lever was illuminated for 20 s. After this, every lever press on the previously active lever resulted in a 20 s illumination of the previously active lever light. Inactive lever presses continued to have no consequence. Twenty-four hours after their seeking test, rats were euthanized via rapid decapitation; mPFC tissue and cecal contents were collected and flash-frozen on dry ice. Rats were dropped from the study if they had a catheter failure, or if they did not average more than 5 infusions over the last 3 sessions of self-administration. Final subject size for this experiment was *n* = 20 H_2_O (10 males, 10 females) and 21 Abx (12 males, 9 females).

### 16S sequencing

Microbial DNA was isolated from the cecal contents of all self-administration rats using Qiagen DNeasy PowerSoil Pro kit per kit instructions. DNA concentration was determined with a NanoDrop1000. One female H_2_O sample was excluded from further analysis due to low DNA concentration. PCR amplification was achieved using primers (341F/805R) targeting the V3 and V4 region of the 16S rRNA region of the bacterial genome and was sequenced on an Illumina NovaSeq (2 × 250 bp paired-end). Amplicons were chimera filtered, dereplicated, and paired-ends were merged using Divisive Amplicon Denoising Algorithm 2 (DADA2). These features were used to determine observed taxonomic units (OTU), defined as sequences with ≥ 97% similarity. OTU counts were used to determine the Shannon index of alpha diversity, the Simpson index, and principle coordinates analysis plots were generated using the Unifrac distance as an assessment of beta diversity using QIIME2 software. OTUs were identified by comparing their genetic sequences to reference bacterial genomes using SILVA (Release 132) with confidence set at 0.7.

### Sample preparation for LC–MS/MS

Medial prefrontal cortex tissue from self-administration rats was lysed with a probe sonicator in solubilization buffer (RIPA buffer containing 1% proteinase and phosphatase inhibitors). Lysate was centrifuged at 14K rpm for 10 min at 4 °C in a tabletop centrifuge to pellet cellular debris, and supernatant was protein precipitated using a methanol chloroform method. Proteins pellet (50 µg) was resuspended in 50uL solubilization buffer (8M urea in 0.4M ammonium bicarbonate), reduced with 5 µL of 45 mM dithiothreitol (DTT) and incubated at 37 °C for 30 min. They were then alkylated with 5 µL of 100 mM iodoacetamide (IAN) and incubated in the dark at room temperature for 30 min. After diluting with water to bring urea concentration to 2 M, sequencing-grade Lys-C (New England Biolabs, Ipswich, MA, USA) was added at a weight ratio of 1:50 (Lys-C/protein) and incubated at 37 °C overnight. Trypsin (Promega, Madison, WI, USA) was then added at similar weight ratio and incubated for 4 h at 37 °C. The digested samples were then acidified with 0.1% formic acid, desalted using C18 spin columns (The Nest Group, Inc., Southborough, MA, USA), and dried in a rotary evaporator. The samples were resuspended in 0.2% trifluoroacetic acid (TFA) and 2% acetonitrile (ACN) in water prior to LC–MS/MS analysis.

### Data-independent acquisition (DIA)

DIA LC–MS/MS was performed using a nanoACQUITY UPLC system (Waters Corporation, Milford, MA, USA) connected to a Q-Exactive HFX (ThermoFisher Scientific, San Jose, CA, USA) mass spectrometer. After injection, the samples were loaded into a trapping column (nanoEase M/Z Symmetry C18 Trap column, 180 µm × 20 mm) for 3 min at a flow rate of 10 µL/min and separated with a C18 column (nanoEase M/Z column Peptide BEH C18, 75 µm × 250 mm). The compositions of mobile phases A and B were 0.1% formic acid in water and 0.1% formic acid in ACN, respectively. The peptides were separated and eluted with a gradient extending from 6% to 25% mobile phase B in 98 min and then to 85% mobile phase B in additional 5 min at a flow rate of 300 nL/min and a column temperature of 37 °C. Column regeneration and up to three blank injections were carried out in between all sample injections. The data were acquired with the Q-Exactive HFX mass spectrometer operating in a data-independent acquisition mode with an isolation window width of 10 m/z. The full scan was performed in the range of 400–1,000 m/z with “Use Quadrupole Isolation” enabled at an Orbitrap resolution of 120,000 at 200 m/z and automatic gain control (AGC) target value of 3 × 10^6^. Fragment ions from each peptide MS^2^ were generated in the C-trap with higher-energy collision dissociation (HCD) at a normalized collision energy of 28% and detected in the Orbitrap at a resolution of 30,000.

DIA spectra were searched against a *Rattus norvegicus* brain proteome fractionated spectral library generated from DDA LC MS/MS spectra (collected from the same Q-Exactive HFX mass spectrometer) using Scaffold DIA software v. 2.2.0 (Proteome Software, Portland, OR, USA). Within Scaffold DIA, raw files were first converted to the mzML format using ProteoWizard v. 3.0.11748. The samples were then aligned by retention time and individually searched with a mass tolerance of 10 ppm and a fragment mass tolerance of 10 ppm. The data acquisition type was set to “Non-Overlapping DIA,” and the maximum missed cleavages was set to 2. Fixed modifications included carbamidomethylation of cysteine residues (+57.02). Dynamic modifications included phosphorylation of serine, threonine, and tyrosine (+79.96), deamination of asparagine and glutamine (+0.98), oxidation of methionine and proline (+15.99), and acetylation of lysine (+42.01). Peptides with charge states between 2 and 4 and 6–30 amino acids in length were considered for quantitation, and the resulting peptides were filtered by Percolator v. 3.01 at a threshold FDR of 0.01. Peptide quantification was performed by EncyclopeDIA v. 0.9.6 and five of the highest quality fragment ions were selected for quantitation. Proteins containing redundant peptides were grouped to satisfy the principles of parsimony, and proteins were filtered at a threshold of two peptides per protein and an FDR of 1%. The mass spectrometry proteomics data have been deposited to the ProteomeXchange Consortium via the PRIDE (Perez-Riverol et al., 2022) partner repository with the dataset identifier PXD079274.

### Pathway analysis and visualization of protein networks

Proteins were excluded from analysis if they were not detected in > 50% of all samples irrespective of treatment. Pairwise comparisons of the Log_10_ median intensity of every remaining protein and protein group were made using Scaffold DIA proteomics analysis software.^1^ Technical replicates were treated as independent samples and proteins were considered significantly differentially regulated when *p* < 0.05. Significantly upregulated and downregulated proteins were separately uploaded into the open source pathway analysis software package G:Profiler (Raudvere et al., 2019)^2^ to identify significantly enriched Gene Ontologies (GO), Kyoto Encyclopedia of Genes and Genomes (KEGG), and Reactome (REAC) pathways using an FDR corrected *p* < 0.05. Given that G:Profiler uses gene names for identifying pathways, all protein names were first converted to gene names prior to analysis using the Uniprot database.^3^ A portion of Figure 1 was made with Biorender.com with full permission to publish.

### Statistical analysis

Rates of active and inactive lever pressing during acquisition and active presses during seeking were calculated as the number of lever presses that occurred during a session divided by the length of the session (90 min for acquisition and 30 min for each portion of the seeking task). Infusions earned, average active lever press rate, and average inactive lever press rate were separately analyzed using linear mixed effects with drink and session as factors with Greenhouse Geiser correction. Percent average self-administration rate during the seeking task was calculated as: [Rate of lever pressing during seeking]/[average rate of lever pressing over the last two sessions of acquisition] × 100. Percent average self-administration rate during context and cue portions of the seeking task were analyzed using two-tailed independent *t*-tests and percent responding during the cue portion of the seeking task was analyzed using a two-tailed Welch’s *t*-test due to group differences in standard deviation. To assess cumulative lever pressing over time during the context and cue portions of the seeking task, the best fit lines for H_2_O and Abx rats were compared using linear regression. Comparison of lever pressing between context and cue portions and lever pressing in response to cue presentation were separately analyzed using two-way mixed ANOVAs. In the event of significant interactions, Sidak’s multiple comparison tests were performed.

To determine differences in the microbiome of Abx rats, alpha diversity was determined using Chao1 and Simpson’s indices. Chao1 was analyzed using a two-tailed independent *t*-test and Simpson’s index was analyzed using a two-tailed Welch’s *t*-test due to groups having unequal standard deviation. Analysis of beta diversity used PERMANOVA tests with 999 permutations using the R statistical package vegan.^4^ Pairwise differences in phyla abundance between H_2_O and Abx rats were compared using Sidak’s multiple comparison test. For all tests, statistical significance was set at *p* < 0.05.

## Results

### Brief Abx during fentanyl self-administration reduces context-induced drug seeking and alters the pattern of responding

There was no effect of Abx on active fentanyl self-administration (Figure 1B). There was a significant effect of session on infusions earned (session: *F*(3.229, 125.6) = 52.63, *p* < 0.0001; drink: *F*(1, 39) = 0.5517, *p* = 0.4621; interaction: *F*(9, 350) = 0.405, *p* = 0.9322) and a significant effect of session for average rate of active lever presses (Figure 1C, session: *F*(3.262, 127.2) = 53.55, *p* < 0.0001; drink: *F*(1, 39) = 0.445, *p* = 0.5087; interaction: *F*(9, 351) = 0.415, *p* = 0.9269). For average rate of inactive lever presses there was a significant effect of session and a significant interaction (session: *F*(3.79, 147.8) = 4.788, *p* = 0.0014; drink: *F*(1, 39) = 0.5997, *p* = 0.4434; interaction: *F*(9, 351) = 2.11, *p* = 0.0281). However, post hoc analysis did not identify a significant treatment effect on any individual day (Figure 1C, all *p* > 0.05).

Rats previously on Abx had reduced percentage active lever pressing only during the context portion of the seeking task (Figures 2A,B for context portion: *t*(39) = 2.036, *p* = 0.044; for cue portion: *t*(39) = 0.376, *p =* 0.709). Since there was only an effect of Abx during the context portion, we next analyzed the pattern of lever pressing across the whole seeking session. We found a trend for Abx rats lever pressing more during the cue portion of the seeking task (Figure 2D, *t*(33.69) = 1.83, *p* = 0.0761). Additionally, analysis of cumulative lever presses over time identified a significant difference between H_2_O and Abx rats during the context portion of the task (Figure 2C, slope H_2_O = 0.4091, slope Abx = 0.2374, *F*(1, 7,376) = 51.56, *p* < 0.0001) but not during the cue portion of the task (slope H_2_O = 0.0827, slope Abx = 0.0770, *F*(1, 7,376) = 0.02421, *p* = 0.8764).

**Figure 2:**
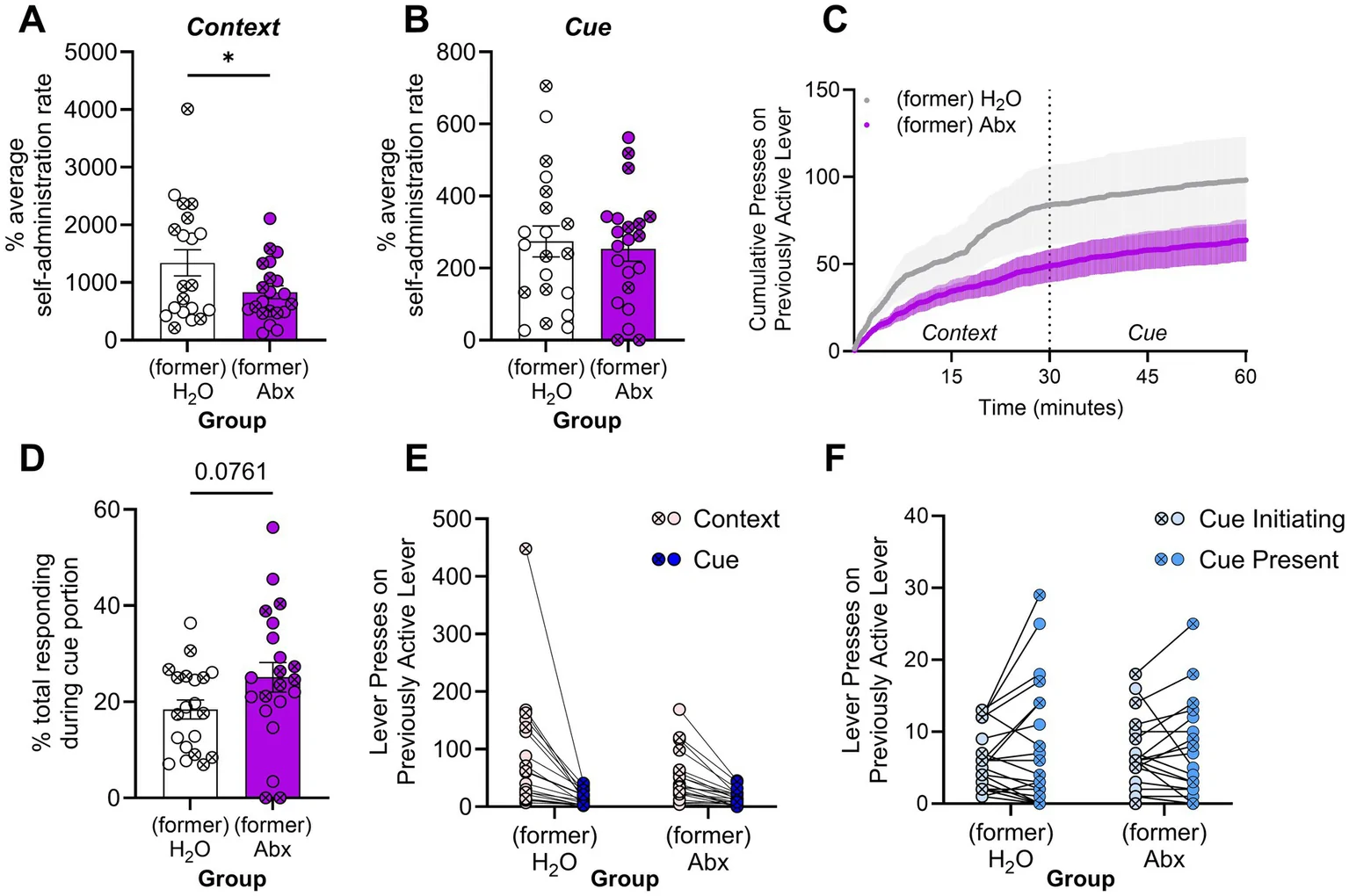
Prior Abx treatment reduces fentanyl seeking and cumulative lever pressing over time. **(A)** Rats formerly on Abx have lower percent response rates during the context portion of the seeking task. **(B)** Former Abx treatment did not affect the percent self-administration rate during the cue portion of the seeking task. **(C)** Cumulative lever presses on previously active lever throughout the seeking task (slope H_2_O = 0.4091, slope Abx = 0.2374, *p* < 0.0001). **(D)** Former Abx almost increased the percent of responses occurring during the cue portion of the task (*p* = 0.0761). **(E)** There was no effect of treatment on previously active lever presses during context and cue portions of the seeking task. **(F)** Treatment had no effect on lever pressing to initiate cues during the cue portion of the seeking task. *p < 0.05. Empty circles are male rats, circles with × are female.

Because prior Abx did not reduce responding during the cue portion of the task despite reducing context-induced seeking, we further assessed whether rats differed in their response to cue presentation during the seeking task. All but one rat lever pressed more during the context portion of the task than the cue portion- indicated by a significant effect of task portion (Figure 2E, task portion: *F*(1, 39) = 22.84, *p* < 0.0001; drink: *F*(1, 39) = 1.593, *p* = 0.2144; interaction: *F*(1, 39) = 2.726, *p* = 0.1067). Next, we wanted to determine if former Abx treatment altered lever pressing during cue light illumination. Former Abx treatment did not affect lever pressing in response to cue presentation (Figure 2F, cue presence: *F*(1, 39) = 1.761, *p* = 0.1922; drink: *F*(1, 39) = 0.1959, *p* = 0.6605; interaction: *F*(1, 39) = 1.313, *p* = 0.2588).

### Brief Abx has long-lasting effects on microbiome composition

Cecal contents were collected 24 h after rats’ seeking test to assess potential differences in the microbiome of rats previously treated with H_2_O or Abx. Despite cessation of Abx two weeks before sample collection, Abx rats had significantly lower microbiome richness, assessed using Chao1 index (Figure 3A, *t*(38) = 13.10, *p* < 0.0001) and had significantly lower evenness (Figure 3B, Simpson’s index: *t*(3.973) = 22.71, *p* = 0.0006), together indicating a reduction in microbiome diversity. The microbiome of former H_2_O rats were significantly distinct from the microbiome of former Abx rats (Figure 3C, treatment effect: *F*(1, 36) = 13.8067, *p* < 0.0001). Further analysis investigated changes at the phyla level. As seen in our prior work using the same Abx cocktail, Abx rats had a reduction in Firmicutes and an expansion in Proteobacteria and Verrucomicrobia (Hofford et al., 2024; Meckel et al., 2024). Additionally, Abx rats showed significant decreases in the proportions of Desulfobacterota, Patescribacteria, Deferribacterota, and unclassified (Figure 3D).

**Figure 3:**
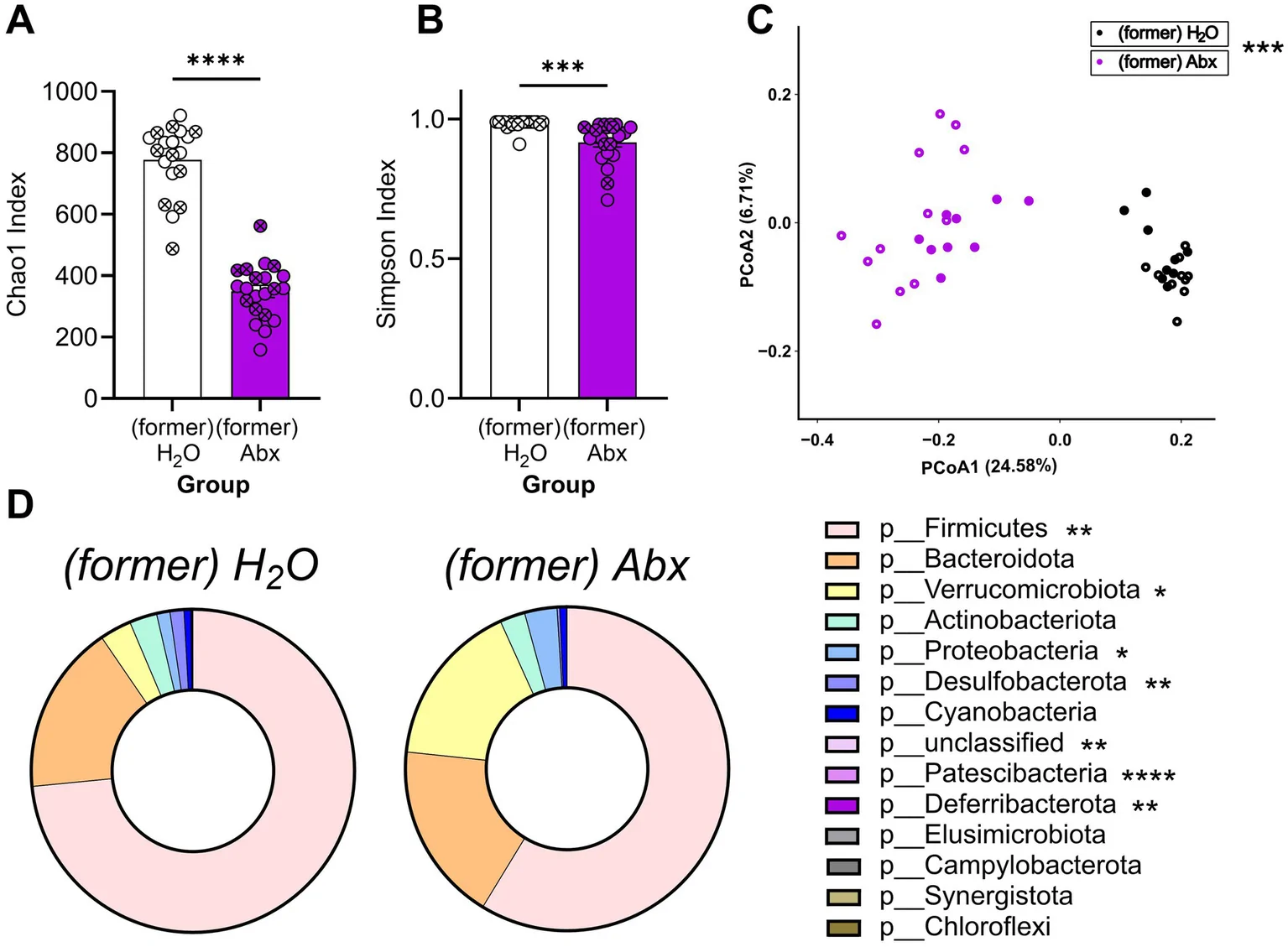
Prior Abx treatment has long-lasting effects on microbiome composition. **(A)** Former Abx treatment reduces alpha diversity as measured by the Chao1 index and **(B)** Simpson index. **(C)** Former treatment results in distinct microbiome clustering as measured using the principle coordinate analysis of unweighted Unifrac distance. **(D)** The percentage of several phyla differ between former Abx treated rats and former H_2_O treated rats. *p < 0.05, **p < 0.01, ***p < 0.001, ****p < 0.0001. Empty circles are male rats, circles with × or solid fill **(C)** are female.

### Brief Abx during adolescent fentanyl self-administration produces long-term effects on mPFC protein expression

Finally, we wanted to explore potential neurobiological changes occurring in rats formerly treated with Abx. To do this, mPFC samples from self-administration rats were processed using data independent acquisition mass spectrometry to assess changes in the proteome. Brief Abx treatment during fentanyl self-administration significantly altered expression of 201 proteins—93 were downregulated and 108 were upregulated (Figure 4A). Pathway analysis using downregulated proteins yielded only two significant pathways—“cellular component organization” and “integrin alphav-beta8 complex.” In contrast, upregulated proteins generated 255 significant pathways. Of these pathways, 13 pathways involved metabolism, which included the prefix “metabol-,” “glycol-,” or included the word “gluconeogenesis” (top metabolism pathways shown in Figure 4B). Additionally, 16 functional pathways (excluding GO:CC) involved neurotransmitter recycling or release. Pathways included the words “neurotransmitter,” “vesicle,” or “exocytosis” (top neurotransmitter pathways shown in Figure 4C).

**Figure 4:**
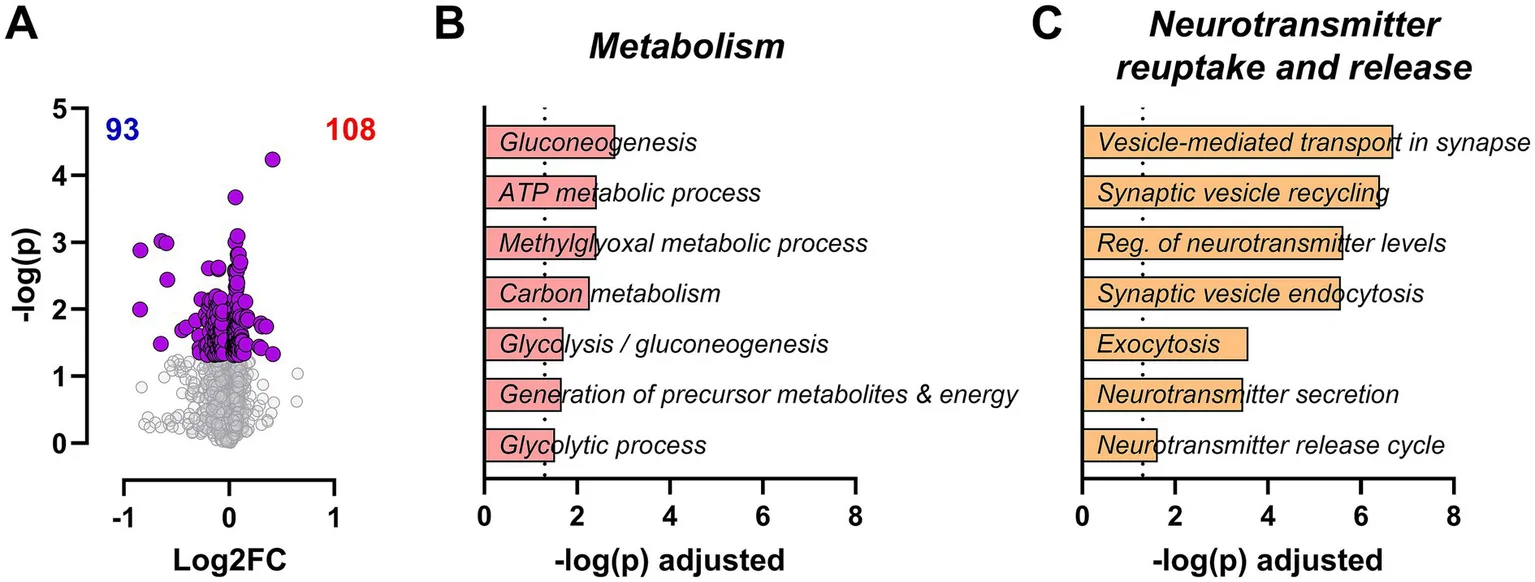
Prior Abx treatment has long-lasting effects on mPFC protein expression. **(A)** Former Abx treatment alters protein expression by causing upregulation of 108 and downregulation of 93 proteins in mPFC. **(B)** Pathway analysis of upregulated proteins identified pathways involved in Metabolism and **(C)** Neurotransmitter reuptake and release.

## Discussion

In this collection of studies, we observed long-lasting impacts of adolescent Abx across all three measured outcomes (fentanyl seeking after abstinence, microbiome composition, and protein expression in mPFC). Prior treatment with Abx reduced context-induced fentanyl seeking (Figure 2A)—a finding unique to this experiment, as our prior work found that Abx treatment increases cocaine and fentanyl seeking in adult rats (Hofford et al., 2024; Meckel et al., 2024). This could be unique to adolescence. However there are other meaningful differences in the design of the current experiment that could be driving reduced seeking including the total Abx duration (10 days versus 1.5 months) and the Abx timing (given during active self-administration only versus throughout self-administration and abstinence). Nevertheless, brief Abx during adolescent self-administration produced the opposite directional change in seeking compared to our prior results in adults. Additionally, the fact that prior Abx only affects context-induced seeking is interesting and might align with our prior work that found Abx reduced opioid CPP in adolescents, but not adults. CPP relies upon associative learning- the association between contextual cues and drug effects are crucial for expression of place preference. Hence, one explanation for both the prior CPP and the current results are that Abx exposure during adolescence impairs contextual learning. Interestingly, the group differences in drug seeking did not persist into the cue portion of the seeking task. While a less likely explanation of the group differences between context- but not cue-induced seeking, it is possible that Abx influences how adolescent rats respond to discrete cues. We explored this further by examining cue responsivity. First, we found that all but one rat responded less during the cue portion of the seeking task compared to the context portion (Figure 2E), indicating that Abx had no effect on this measure. Second, Abx did not influence the number of responses rats exerted to initiate a cue or the number of responses rats exerted in the presence of the cue light and there was no interaction between response type and treatment (Figure 2F). Together, these negative findings fail to support the hypothesis that prior Abx enhances cue responsivity. However, the design of this task has an inherent confound in that the context portion preceded the cue portion. To truly answer the question of altered cue responsivity after adolescent Abx, studies utilizing Pavlovian instrumental transfer would have to be conducted (Cartoni et al., 2016). Given a lack of support for enhanced cue responsivity, it is possible that Abx causes a delay in responding overall reflected by a higher proportional responding during the later portion of the task.

A possible explanation for the current behavioral findings is that Abx treatment during active self-administration produced a unique internal state that differed from the internal state present during seeking (since rats were no longer on Abx). This could have resulted in lower context-induced seeking in Abx rats since they were experiencing an internal state mismatch compared to rats that remained on H_2_O for the whole experiment. The experiments conducted in the current manuscript answered the question of whether or not adolescent microbiome disturbance affects behavior in young adulthood. Thus, it was not designed to test whether Abx given during self-administration produced state-dependent learning. Future studies could compare fentanyl seeking in rats that remained on their drinks for the entire experiment (H_2_O throughout and Abx throughout) to rats that switch drinks during abstinence (Abx to H_2_O and H_2_O to Abx) to support or refute this conclusion.

We also observed long-lasting alterations in the composition of the gut microbiome after Abx treatment ended. The phyla changes observed are similar to what we have observed previously when animals are on Abx for at least 2 weeks, with a reduction in Firmicutes and an expansion of Proteobacteria and Verrucomicrobia (Hofford et al., 2021, 2024; Meckel et al., 2024). The magnitude of diversity reduction and its duration was surprising, since an earlier adolescent mouse study that administered Abx for 10 days immediately before tissue collection observed a much smaller reduction in alpha diversity (Hofford et al., 2025). Unfortunately, we cannot definitively determine whether microbiome alterations restricted to adolescence truly have enduring effects on behavior and mPFC protein expression, since the microbiome of H_2_O and Abx rats still differed so much by the end of testing. However, this does indicate that brief Abx has long-lasting effects on the microbiome that do not return to baseline in the absence of interventions (e.g., transfer of dirty bedding between cages or fecal microbiota transplant).

Finally, brief exposure to Abx during adolescence altered the mPFC proteome. This study builds on our prior work that identified robust changes in mPFC gene expression networks after adolescent Abx and morphine (Hofford et al., 2025). Like the previous study, animals exposed to Abx and fentanyl show altered expression patterns than those exposed to just H_2_O and fentanyl, with the current study finding 201 differentially expressed proteins in Abx rats compared to H_2_O rats. A head-to-head comparison of pathways identified from RNA sequencing and proteomics do not seem to overlap between the prior study and the current one. Our previous work identified pathways involved in gene transcription, chromatin modification, and development (Hofford et al., 2025), while the current proteomic pathways involve general metabolism and neurotransmitter release and recycling. Yet some technical details preclude a meaningful comparison between the RNA sequencing results and proteomics. Proteomics relies upon a comparison of identified peptides against specific protein libraries. This study referenced a neuronal synaptic library- thus primarily identifying proteins in neuronal cell bodies, dendrites, and axon terminals which would not capture changes in nuclear proteins or changes in other cell types. Nevertheless, protein expression changes are a more reliable read-out of functional changes than transcriptomics, since results from RNA-sequencing poorly correlate with protein changes (Ghazalpour et al., 2011; Schwanhäusser et al., 2011). One limitation of the current experimental design is the lack of saline/H_2_O and saline/ Abx groups. Because we lack these comparison groups, we are unable to determine the effects of Abx or fentanyl alone on mPFC protein expression.

Taken together, these findings suggest that brief antibiotic (Abx) exposure during adolescence produces lasting alterations in drug seeking, microbiome composition, and mPFC neurobiology that extend at least two weeks after Abx cessation. These effects could be adolescent specific, consistent with evidence that brain development, signaling, and behavior are susceptible to adolescent microbiome disturbance (Sudo et al., 2004; Desbonnet et al., 2015; Al Nabhani et al., 2019; Hofford et al., 2025) and with our prior observation of the opposite behavioral effects in adult rats (Hofford et al., 2024; Meckel et al., 2024). However, given the long-lasting effects of brief Abx on the microbiome, additional studies are needed to determine whether this is truly an adolescent-specific phenomenon. Nevertheless, this study demonstrates that brief Abx exposure during adolescence can have long-lasting consequences on both brain and behavior, with implications for understanding how early-life microbiome perturbations shape vulnerability to substance use disorders.

## Data Availability

The datasets presented in this study can be found in online repositories. The names of the repository/repositories and accession number(s) can be found at: https://www.ebi.ac.uk/pride/archive/, PXD079274.

## Footnotes

1
http://www.proteomesoftware.com/products/dia/

2
https://biit.cs.ut.ee/gprofiler/gost

3
https://www.uniprot.org/id-mapping

4
http://github.com/vegandevs/vegan
